# Modulation of salivary cytokines in response to alcohol, tobacco and caffeine consumption: a pilot study

**DOI:** 10.1038/s41598-018-35094-z

**Published:** 2018-11-12

**Authors:** Chirag C. Sheth, Rosa M. López-Pedrajas, Maria del Mar Jovani-Sancho, Raquel González-Martínez, Veronica Veses

**Affiliations:** 1 Department of Medicine, Faculty of Health Sciences, Universidad CEU Cardenal Herrera, CEU Universities, Moncada, 46113 Valencia, Spain; 2 Department of Biomedical Sciences, Faculty of Health Sciences, Universidad CEU Cardenal Herrera, CEU Universities, Moncada, 46113 Valencia, Spain; 3 Department of Dentistry, Faculty of Health Sciences, Universidad CEU Cardenal Herrera, CEU Universities, Moncada, 46113 Valencia, Spain

## Abstract

It has been shown that long-term stimulant consumption alters the biological and microbiological status of the oral cavity. We present a pilot study describing stimulant-specific oral immunomodulation in the oral cavity. Changes in salivary cytokine levels in response to long-term alcohol, tobacco and caffeine were identified. Volunteers were recruited from amongst the patients visiting University Dental Clinic of CEU Cardenal Herrera University (Alfara del Patriarca, Spain). Participants were grouped according to their self-reported levels of consumption of either caffeine, alcohol or tobacco (control group volunteers were non-consumers of all three). Informed consent was provided and stimulated saliva samples were obtained and assayed for interleukin-1α IL-1α), Tumor Necrosis Factor α (TNF-α) and Interferon γ IFN-γ). Long-term, high-level consumers of alcohol or tobacco exhibited elevated salivary concentrations of the three inflammatory cytokines with respect to control values. Specifically, IL-1α was found to be elevated in alcohol users whilst IFN-γ concentration higher in tobacco users versus controls. Long-term caffeine consumers displayed elevated levels of IFN-γ and TNF-α, whereas IL-1α levels were reduced with respect to control volunteers. This pilot study demonstrates that salivary cytokines can be modulated in response to quantity and duration of alcohol, caffeine or tobacco consumption.

## Introduction

Cytokines are soluble messenger molecules that mediate the interplay between innate and adaptive immune system^1^. Cytokines have been shown to be biologically active at low concentrations^2^, and play a role in the mediation of local inflammatory reactions. Most cytokines are secreted by immune cells in response to infection, although other types of cells can also produce them^1^. Oral mucosal epithelial cells produce a variety of cytokines in a constitutively manner in response to opportunistic oral infections^3–7^. When the levels of oral cytokines are altered, for example by the elevated presence of opportunistic microorganisms, inflammation can become chronic, leading to oral pathologies including cancer and periodontal disease^8,9^.

Previous studies have highlighted the role played by pro-inflammatory cytokine interleukin-1alpha (IL-1α), tumor necrosis factor-alpha (TNF-α) and interferon-gamma (IFN-γ) in disease processes in the oral cavity.

Elevated salivary IL-1α levels have been shown to be associated with oral squamous cell carcinoma^10^, erosive oral lichen planus^11,12^, bisphosphonates-related osteonecrosis of the jaws^13^, and in response to microbial colonization^6^.

Increased salivary TNF-α concentrations often appear in conjunction with elevated IL-1α in saliva. Analysis of saliva samples from patients with periodontitis^14^, burning mouth syndrome^15^, recurrent aphthous stomatitis^16^ and in response to microbial colonization^6^ support the role of TNF-α as a link between inflammation and disease.

The central role of IFN-γ in the defense against viral and intracellular bacterial infections is well studied^17^. We include the examination of this key cytokine due to its role in innate and adaptive immunity, highlighted by the elevated susceptibility of long-term alcohol, and tobacco consumers to viral infections leading to cancers of the oral cavity^18–20^. IFN-γ is produced by and stimulates a wide array of immune cells, including NK (natural killer), NKT (natural killer T), CD4+ and CD8+ T cells, leading to the downstream modulation of other pro-inflammatory cytokines, such as TNF-α^16^. The balance between pro and anti-inflammatory cytokines is essential for maintenance of health.

It is well known that alcohol and tobacco consumption have a strong negative impact on oral health^21–23^. The association between smoking and periodontal disease has been recognized for a long time, with a 1.4 to five-fold increased relative risk for periodontitis among smokers^24–26^. The relationship between smoking and oral cancer has been fully characterized^27^. Aromatic hydrocarbon benz-pyrene and nitrosamines in tobacco smoke induce mutations in DNA replication, initiating neoplastic changes in the oral mucosa^28^. Alcohol consumption has been reported as a leading cause of oral lesion development^29^. Furthermore it has been shown to act synergistically with tobacco in increasing the risk of development of oral cancer^28^.

Effects of other stimulants such as caffeine in the oral cavity are controversial. Some studies describe an increment in inflammatory activity as a result of caffeine consumption whilst others describe the anti-inflammatory effects of caffeine in the oral mucosa^30,31^.

Published research, including work from our group has shown that long-term stimulant consumption plays a role in the modification of the level of colonization by key opportunistic pathogens in a species-specific manner^32–34^. Published evidence suggests that perturbation of components of the oral microflora (keystone pathogens) may lead to dysbiosis and the unbalancing of the host-microbial interactions, favoring the disease state^35^.

Previous published work from our group suggests a link between stimulant use and perturbation of normal oral microflora^34^. This pilot study seeks to investigate whether changes in oral cytokine levels (IL-1α, TNF-α, and INF-γ) are associated with stimulant consumption.

## Results

In order to calculate the post-hoc power of the study, the Pillai-Bartlett V criterion was set at 0,4 including 4 study groups each represented by 3 response variables (Table 1). Volunteers were clinically examined to assess their oral health status. Individuals with periodontitis, oral lesions, active infection foci or DMFT indices ≥4.4 were excluded from the study. Volunteers admitted to the study were therefore orally healthy by clinical examination.

**Table 1:** Distribution of the study volunteers into study groups based on the duration and quantity of consumption of alcohol tobacco and caffeine.

Study group	Duration	Quantity	Reference
Alcohol consumers	>10 years (Long-term)	• ≥4 units (40 g of ethanol) per day for males • ≥3 units (30 g of ethanol) per day for females • Non consumer of tobacco or caffeine	^36^
Tobacco consumers	>10 years (Long-term)	• ≥20 cigarettes/day; each cigarette containing 2 g of tobacco) • Non-consumer of alcohol or caffeine	^37^
Caffeine consumers	>10 years (Long-term)	• ≥600 mg/day of coffee, tea or caffeinated beverages • Non-consumer of alcohol or tobacco	^38^
Control	—	• Non-consumer of alcohol, caffeine or tobacco	—

Individuals allocated to the “Alcohol consumers” group were those consuming high daily quantities of alcohol^36^ habitually for longer than 10 years, with no consumption of caffeine or tobacco. Volunteers assigned to the “Tobacco consumers” group were individuals consuming high quantities of tobacco^37^ daily for 10 years or more, in addition to no consumption of the other two stimulants. The study participants forming part of the “Caffeine consumers” group included individuals with a daily consumption of ≥600 mg of caffeine (as coffee, tea or caffeinated beverages) for a minimum period of 10 years^38^. These volunteers did not consume alcohol nor tobacco. The control group was composed of volunteers who did not consume any of the studied stimulants.

The calculated effect size f^2^(V) was found to be 0.1538. Setting the α probability of error to 0,5 and a total sample size of 44 volunteers, the power of this study was calculated as being 88%, which we believed to be highly satisfactory, given the pilot nature of the study.

Of the 44 healthy volunteers included in this study, 9 were long-term, high consumers of tobacco (20.5%), 9 were long-term high consumers of alcohol (20.5%). Nine individuals (20.5%) were long-term high consumers of caffeine, and 17 control individuals (38.6%) were included (Table 2). The mean age of the control volunteers was 36.8 ± 4.8 years, 43.7 ± 2.3 years for alcohol consumers, 42.1 ± 5.1 years for tobacco consumers, and 31.8 ± 1.9 years for caffeine consumers (Table 2). All volunteers included in the study received their last dental check-up and cleaning between 2 to 6 months prior to the collection of the saliva sample (Table 2).

**Table 2:** Demographic characteristics of the population of study volunteers, including the sex distribution, mean age and date of last dental checkup.

	Control	High alcohol	High tobacco	High caffeine
Male % (n)	29,4 (5)	66,7 (6)	55,6 (5)	44,4 (4)
Female % (n)	70,6 (12)	33,3 (3)	44,4 (4)	55,6 (5)
Mean Age in years (SE)	36,8 (4,8)	43,7 (2,3)	42,1 (5,1)	31,8 (1,9)
Last dental checkup	2–6 months prior to study			

The Shapiro-Wilk test was applied to determine whether the observed pattern of cytokine expression was normally distributed. A p-value > 0.05 indicates that the data follow a normal distribution. In order to apply standard parametric significance tests, all data must be normally distributed. The results of the Shapiro-Wilk test (Table 3) indicate that all data were not normally distributed.

**Table 3:** Results of the Shapiro-Wilk normality test, demonstrating the normal (bold, italic) and non-normal (italic) distributions.

	Group	Shapiro-Wilk		
		Statistic	gl	Sig.
IFN-γ	Control	,942	15	*,* ***407***
	High alcohol	,694	7	*,003*
	High caffeine	,899	7	*,* ***324***
	High Tobacco	,654	7	*,001*
TNF-α	Control	,741	15	*,001*
	High alcohol	,497	7	*,000*
	High caffeine	,906	7	*,* ***369***
	High Tobacco	,783	7	*,028*
IL-1α	Control	,875	15	*,040*
	High alcohol	,874	7	*,* ***203***
	High caffeine	,899	7	*,* ***322***
	High Tobacco	,830	7	*,* ***080***

### Cytokine profile of IFN-γ in response to stimulant consumption

Baseline levels of IFN-γ were established in the saliva of healthy volunteers, non-consumers of stimulants (neither alcohol, tobacco, nor caffeine). Median values found in the control group were 8.1 pg/ml (Standard deviation (SD) 5.6 pg/ml; Table 4). In habitual consumers of alcohol, this value was found to be 12.9 pg/ml (SD = 18.4 pg/ml), whilst the values for caffeine and tobacco were 8.3 (SD = 14.7 pg/ml) and 13.8 (SD = 23 pg/ml) respectively (Table 4).

**Table 4:** Descriptive analysis of the modulation of the three cytokines in control participants and volunteers consuming alcohol, tobacco and caffeine.

		GROUP			
		Control	High alcohol	High caffeine	High Tobacco
IFN-γ	Participants per group (*n)*	16	7	9	8
	Mean	8,72	15,81	12,45	21,26
	Standard deviation	5,62	18,40	14,71	23,04
	Median	8,08	12,91	8,29	13,84
TNF-α	Participants per group (*n)*	15	7	8	7
	Mean	10,68	48,04	28,81	24,86
	Standard deviation	15,18	113,08	30,93	34,35
	Median	,00	5,40	23,69	,00
IL-1α	Participants per group (*n)*	16	7	7	7
	Mean	149,37	390,99	130,87	202,67
	Standard deviation	124,74	374,34	75,01	231,77
	Median	102,39	193,89	111,88	122,66

### Cytokine profile of TNF-α in response to stimulant consumption

Control patients were found to express a baseline median salivary TNF-α concentration of 0 pg/ml (SD = 15.2 pg/ml; Table 4). The median concentration determined for alcohol consumers was 5.4 pg/ml (SD = 113 pg/ml), for the caffeine consumers, this value was 23.7 pg/ml (SD = 30.9 pg/ml), whilst for tobacco consumers, the median value was found to be 0 pg/ml (SD = 34.35 pg/ml) (Table 4).

### Cytokine profile of IL-1α in response to stimulant consumption

The median salivary concentration of IL-1α in control patients was found to be 102 pg/ml (SD = 124.7 pg/ml; Table 4). In volunteers habitually consuming high levels of alcohol, the median salivary concentration was ascertained as being 193.9 pg/ml (SD = 374 pg/ml) whilst the corresponding values in caffeine and tobacco consumers were determined as 111.9 pg/ml (SD = 75 pg/ml) and 122.7 pg/ml (SD = 231.8 pg/ml) respectively (Table 4).

### Result of the test for significance (Kruskal-Wallis)

The Kruskal-Wallis test is a non-parametric analysis comparing the distributions of two or more independent samples to determine whether the distribution of cytokines differ significantly between volunteer groups. The p-values of the analysis were found to be greater than 0.05 (IFN-γ p = 0.366, TNF-α p = 0.532 and IL-1α p = 0.364), indicating that the results were not statistically significant.

## Discussion

The widespread, often long-term consumption of stimulants such as tobacco, caffeine and alcohol has been extensively studied for its deleterious effects on general and oral health^21–23,28,30^. Few studies, however, have investigated the combined effects of these substances on salivary immunological markers with their associated impact on the oral microbial flora. The frequent and often combined use of the chosen stimulants made recruitment of volunteers for this study a difficult process. In order to dissect the stimulant-specific oral cytokine modulation intentionally stringent volunteer selection criteria were applied such that participants assigned to each study group were exclusive, high-level, long-term consumers of only one stimulant, or none in the case of the control group.

The male to female ratios in the study groups varied from 0.4:1 to 2:1 (Table 2). Gender-based differences in cytokine profiles were not evaluated in this study as the three salivary cytokines considered have been shown to be unaffected by sex in previous studies^2^. There was no statistically significant difference in mean ages between the four study groups. The similarity in mean age across the groups strengthens the conclusions of this study since aging has been shown to result in immunosuppression^39^.

The strengths of this study include the high level of stringency in patient selection, the selection of orally healthy volunteers, which precludes immunomodulation due to underlying conditions and the robustness of the statistical analysis applied. The study was strongly negatively affected by the small sample size, which was likely to be the main factor resulting in the lack of statistical significance. It is our opinion that in this study, the lack of demonstrated statistical significance should not be considered as the sole factor determining the importance of the data in the clinical context. As published elsewhere^40,41^, it is critical to consider the importance of the results both in the context of clinical significance and absolute difference. The clinical implications of the data from this study, which demonstrate stimulant-specific modulation of salivary cytokines should not be ignored. The relatively small sample size and the limited number of cytokines studied reflect the pilot nature of the study.

Saliva was chosen as it is readily obtained in larger volumes as compared to gingival-crevicular fluid, and is representative of the inflammatory status of the whole mouth^17,42,43^. Salivary cytokine levels have been shown to be altered in a variety of oral conditions, thus justifying the use of this material as a convenient biomarker for oral health^2,44,45^.

Our study revealed an interesting modulation of salivary cytokines, revealing an elevation (albeit not statistically significant) of discrete cytokines associated with each stimulant with respect to control volunteers. Elevated salivary levels of IFN-γ and TNF-α were expressed by individuals who were long-term consumers of each of alcohol, tobacco or caffeine, compared to control volunteers. The reference levels of IFN-γ and TNF-α and IL-1α observed in our study for control volunteers were lower than those published by Khan^2^, however differences in saliva collection methods (whole versus stimulated) may explain the variation. The salivary IFN γ concentration in control volunteers in this study was found to be similar to that obtained by Marques *et al*.^46^, also stimulated saliva. The concentration of TNF-α in stimulated saliva from control volunteers correlated well with data from other studies^47^. We were unable to identify comparative studies in which the concentration of IL-1α was determined in stimulated whole saliva samples. Two studies employing unstimulated saliva samples presented higher concentrations of IL-1α as compared to the present study^2,48^.

The salivary IL-1α concentration in the present study was found to be higher in consistent, long term consumers of alcohol or tobacco and lower in caffeine consumers with respect to control volunteers. IFN-γ is a type II cytokine produced by type 1 T helper (T_h_1) cells, cytotoxic T (T_c_) cells and others including mucosal epithelial cells, in response to viral, bacterial and protozoal infections^49,50^. Studies have shown that TNF-α and IL1-α levels are elevated in patients suffering from oral lichen planus^51,52^, dental caries^53^ and periodontitis^54–58^.

Our results suggest a model in which long term stimulant dependent inflammation^25,59,60^, induces a T_h_1-type response in oral mucosal epithelial cells, resulting in the production of IFN γ. The prolonged induction of IFN γ has been shown to stimulate and activate leukocytes including macrophages and neutrophils, promoting inflammatory cytokine production in the presence or absence of microbial components^61–63^. Our group has previously published evidence to demonstrate the stimulant-specific modulation of microbial colonization^34^. The current study suggests that the long-term stimulant consumption-induced changes in cytokine production may alter local oral immunity thereby facilitating the microbial dysbiosis.

To the best of our knowledge, this pilot study is the first to report specific alterations of oral pro-inflammatory cytokine levels in response to long-term stimulant consumption in orally healthy individuals.

In the light of the above results, we feel that further studies involving greatly increased patient numbers should be carried out to fully characterize changes in oral cytokine production in healthy individuals as a result of stimulant consumption. The impact of these changes on oral microbial colonization, and as a trigger for dysbiosis remains to be evaluated.

## Materials and Methods

### Recruitment of study volunteers

The study protocol received the approval of the Bioethics Committee of Universidad CEU Cardenal Herrera (Reference number CI13/001). Written informed consent was obtained from all study participants.

A total of 44 volunteers were recruited amongst patients attending the Dental Clinic of the Universidad CEU Cardenal Herrera in Alfara del Patriarca (Spain). Inclusion criteria were: orally healthy volunteers, older than 18 years of age, of either sex. Patients were excluded if they had received antibiotic therapy during 60 days prior to enrollment, suffered from diabetes or periodontitis, were immunosuppressed, pregnant or suffered from a systemic disease. The oral cavities of volunteers who signed their informed consent were examined clinically by qualified dentists participating in the research project, in order to determine the number of decayed, missing filled teeth (DMFT index). Volunteers with a DMFT of <4.4^64^ were considered orally healthy and admitted to the study. Additionally, volunteers were asked to complete a questionnaire regarding their stimulant consumption and oral hygiene habits and assigned to the different study groups in accordance with the duration and quantity of alcohol, tobacco and caffeine consumption (Table 1).

### Sample collection

Whole stimulated saliva samples were collected in the morning. All volunteers followed the same procedure for saliva collection. Volunteers had not eaten for 2 hours prior to the study. Patients were asked to chew on a paraffin wax tablet for 1 minute followed by expectoration of the saliva into sterile beakers using the spitting technique, according to previously published protocols^65^. Individual 1 ml aliquots of the saliva samples were transferred to sterile 1.5 ml microfuge tubes and stored at −20 °C for posterior cytokine analysis.

### Cytokine analysis

Salivary cytokine levels were quantified using commercial ELISA kits. Human IL-1 alpha/IL-1F1 DuoSet (R & D Systems, España, Cat # DY200) was used for the determination of Interleukin 1-α. Tumour Necrosis Factor-α levels in saliva were assessed with Human TNF-alpha DuoSet (R & D Systems, España, Cat # DY210). Human IFN-gamma DuoSet (R & D Systems, España, Cat # DY285) kit was used for quantification of Interferon-γ. Samples were analyzed in triplicate, and values were obtained in picogram/ml (pg/ml).

### Statistical analysis

Data were presented graphically using standard Microsoft Excel® software. Mean values and standard deviations were obtained for the salivary cytokines analyzed in the study. The Shapiro-Wilk test was applied to determine whether the observed pattern of cytokine expression was normally distributed. The Kruskall-Wallis (non-parametric) test for comparing 2 or more samples was therefore applied to test whether cytokine expression was significantly different between patient groups. A post-hoc power calculation was performed to determine the resultant power of the study (G*Power^66^).

### Ethical approval

All procedures performed in studies involving human participants were in accordance with the ethical standards of the institutional and national research committee and with the 1964 Helsinki Declaration and its later amendments or comparable ethical standards. The study was approved by the Ethics Committee of CEU Cardenal Herrera University (authorization number CEI13/001).

### Informed consent

Informed consent was obtained from all individual participants included in the study

## Data Availability

The datasets generated during the current study are available from the corresponding author on reasonable request.
